# Performance of a cardiac lipid panel compared to four prognostic scores in chronic heart failure

**DOI:** 10.1038/s41598-021-87776-w

**Published:** 2021-04-14

**Authors:** Peter McGranaghan, Anshul Saxena, Hans-Dirk Düngen, Muni Rubens, Sandeep Appunni, Joseph Salami, Emir Veledar, Philipp Lacour, Florian Blaschke, Danilo Obradovic, Goran Loncar, Elvis Tahirovic, Frank Edelmann, Burkert Pieske, Tobias Daniel Trippel

**Affiliations:** 1 Department of Internal Medicine and Cardiology, Charité – Universitätsmedizin Berlin, Campus Virchow-Klinikum, Augustenburger Platz 1, 13353 Berlin, Germany; 2 Baptist Health South Florida, 6855 Red Rd, Coral Gables, FL 33143 USA; 3 Department of Biochemistry, Government Medical College, Kozhikode, Kerala 673008 India; 4 Department of Cardiology and Internal Medicine, Heart Center Leipzig at the University of Leipzig, Russenstrasse 69A, 04289 Leipzig, Germany; 5 Institute for Cardiovascular Diseases Dedinje, Department of Cardioloy, Faculty of Medicine, University of Belgrade, Heroja Milana Tepića br. 1, 11040 Belgrade, Serbia; 6 Apostolovic Clinic for Cardiovascular Diseases, Clinical Centre Nis, University of Niš, Niš, Serbia; 7 DZHK (German Centre for Cardiovascular Research), Berlin, Germany; 8 Berlin Institute of Health (BIH), Berlin, Germany; 9 Department of Internal Medicine and Cardiology, German Heart Centre Berlin, Berlin, Germany; 10 Department of Biostatistics, Florida International University, Miami, FL USA; 11 Division of Cardiology, Emory University School of Medicine, Atlanta, GA USA

**Keywords:** Prognostic markers, Metabolomics, Molecular medicine

## Abstract

The cardiac lipid panel (CLP) is a novel panel of metabolomic biomarkers that has previously shown to improve the diagnostic and prognostic value for CHF patients. Several prognostic scores have been developed for cardiovascular disease risk, but their use is limited to specific populations and precision is still inadequate. We compared a risk score using the CLP plus NT-proBNP to four commonly used risk scores: The Seattle Heart Failure Model (SHFM), Framingham risk score (FRS), Barcelona bio-HF (BCN Bio-HF) and Meta-Analysis Global Group in Chronic Heart Failure (MAGGIC) score. We included 280 elderly CHF patients from the Cardiac Insufficiency Bisoprolol Study in Elderly trial. Cox Regression and hierarchical cluster analysis was performed. Integrated area under the curves (IAUC) was used as criterium for comparison. The mean (*SD*) follow-up period was 81 (33) months, and 95 (34%) subjects met the primary endpoint. The IAUC for FRS was 0.53, SHFM 0.61, BCN Bio-HF 0.72, MAGGIC 0.68, and CLP 0.78. Subjects were partitioned into three risk clusters: low, moderate, high with the CLP score showing the best ability to group patients into their respective risk cluster. A risk score composed of a novel panel of metabolite biomarkers plus NT-proBNP outperformed other common prognostic scores in predicting 10-year cardiovascular death in elderly ambulatory CHF patients. This approach could improve the clinical risk assessment of CHF patients.

## Introduction

The prevalence of chronic heart failure (CHF) in the western world continues to increase, especially in patients older than 65 years^1^. CHF is a major burden on the health care system and is associated with high morbidity and mortality, including a poor quality of life^2^. An important aspect of CHF management is to ensure that clinicians and patients with CHF have the necessary knowledge and resources to make the best health decisions. A prognostic model is one such resource, defined as a formal combination of multiple predictors from which risks of a specific outcome can be calculated for individual patients.

Prognostic models are abundant in the literature, and the most popular ones include the SHFM (Seattle Heart Failure Model), FRS (Framingham Risk Score), MAGGIC (Meta-analysis Global Group in Chronic Heart Failure), and BCN Bio-HF (Barcelona Bio-Heart Failure Risk Calculator). The SHFM score is the most thoroughly validated and contains the most predictor variables of the four^3^. The MAGGIC score^4^ was developed from a dataset of over 39,000 patients across 30 studies and validated on more than 60,000 patients using 2 large CHF cohorts^5,6^. The FRS score was developed as a sex-specific risk score that can be conveniently used to calculate general cardiovascular disease (CVD) risk and risk of individual CVD events^7^. The BCN Bio-HF score contains 11 clinical variables with the most biomarker variables [NT-proBNP, high-sensitivity cardiac troponin T (hs-cTnT), high-sensitivity soluble ST2 (ST2)] and has been externally validated^8,9^. These models all use common clinical and demographic variables to predict the prognosis of CHF patients and have convenient online calculators. Although these scores have been validated, they have not been widely adopted possibly because they are not routinely calculated in clinical practice^10–12^, have poor reliability at the individual patient level^5^, suffer from a significant amount of missing data requiring imputation.

Metabolomics is a rapidly growing field in biomarker profiling that could help meet the need for more robust prognostic biomarkers. By applying nuclear magnetic resonance (NMR) spectroscopy and mass spectrometry (MS), it is now possible to analyze hundreds of metabolites from human samples such as blood, urine, saliva, and tissue, which can elucidate the outcome of complex networks of endogenous and exogenous biochemical reactions^13^. This approach could provide a more comprehensive signature of biochemical activities that could be associated with diet, medication, disease progression, and thus negative outcomes due to these complex mechanisms^14,15^. Previous studies have shown that metabolomic biomarkers can be used for risk prediction as well as diagnosis of CHF^16–28^.

One promising metabolomic biomarker panel in CHF patients is the cardiac lipid panel (CLP) which is supplemented by N-terminal pro–B-type natriuretic peptide (NT-proBNP). The CLP is consists of three specific metabolomics features: triacylglycerol (TAG) 18:1/18:0/18:0, phosphatidylcholine (PC) 16:0/18:2, and the sum of the 3 isobaric sphingomyelin (SM) species SM d18:1/23:1, SM d18:2/23:0, and SM d17:1/24:1. The diagnostic value of CLP was first discovered in a study by Mueller and colleagues, where they compared CHF patients to healthy controls, and found that CLP was able to improve the diagnostic performance over NT-proBNP alone^29^. The incremental prognostic value of the CLP was first assessed in a recent study which found it improved the discrimination and risk assessment over NT-proBNP and clinical risk factors^30^.

The objective of this study was to compare the performance of a risk score composed of the CLP panel plus NT-proBNP to the four commonly used traditional risk scores (SHFM, FRS, MAGGIC, BCN Bio-HF) to predict long-term cardiovascular mortality in ambulatory CHF patients. We hypothesized that the CLP risk score would improve our ability to classify risk of cardiovascular death in comparison to the four validated clinical risk prediction algorithms.

## Results

Table 1 shows the baseline characteristics of the total population (n = 280) as well as the variables included in each score. Mean age of this sub-cohort was 72.1 (4.9) years, 26.4% were women, 45% patients had heart failure with reduced ejection fraction (HFrEF) (LVEF < 35%), and most patients were in NYHA functional class II (67.5%) with the remaining in NYHA class III. Hypertension was present in 80% of participants and 45% were current or former smokers; 29% had diabetes and 71% had CAD. During the follow-up period (mean = 81 months, SD = 33; median = 96 months), 95 (34%) patients met the primary outcome. There were 30 (11%) patients who met the secondary outcome of 3-year all-cause mortality. The sample selection criteria as well as the comparison of this sub cohort’s baseline characteristics to the source cohort has previously been reported^30^, however, this study analyzed 10 year follow up rather than the previously reported 4 year follow up.

**Table 1:** Baseline characteristics of the study participants and variables included in each prognostic score.

Characteristic	Total	Prognostic score				
	n = 280	SHFM	FRS	MAGGIC	BCN Bio-HF	CLP
Age (years), mean ± SD	72 ± 4.9	✓	✓	✓	✓	
NYHA (II/III), n	188/91	✓		✓	✓	
Male, n (%)	206 (74)	✓	✓	✓	✓	
Body mass index (kg/m^2^), mean ± SD	26.8 ± 3.4			✓		
Heart rate (bpm), mean ± SD	73 ± 13.0					
Systolic blood pressure (mm Hg), mean ± SD	134 ± 19	✓	✓	✓		
Diastolic blood pressure (mm Hg), mean ± SD	81 ± 11					
Years since first diagnosis of CHF	5.2 ± 5.6		✓			
**Laboratory, mean ± SD**						
Creatinine (μmol/L)	107 ± 27.9			✓		
Hemoglobin (g/dL)	13.4 ± 1.5	✓			✓	
Sodium (mEq/L)	141.4 ± 3.3	✓			✓	
Uric acid (μmol/L)	356 ± 127	✓				
Total Cholesterol (mmol/L)	5.1 ± 1.6					
HDL cholesterol (mmol/L)	1.2 ± 0.5		✓			
LDL cholesterol (mmol/L)	3.4 ± 1.3					
Triglycerides (mmol/L)	1.7 ± 1.0					
Lymphocytes (%)*		✓				
NT-proBNP (pg/mL)	793 (331–1765)^†^				✓	✓
PC 16:0/18:2 (µg/dl)	36,810 (32,435–40,015)^†^					✓
TAG 18:1/18:0/18:0 (µg/dl)	121 (76.5–256.4)^†^					✓
SM d18:1/23:1, SM d18:2/23:0, SM d17:1/24:1 (µg/dl)	1342 (1134–1596)^†^					✓
**Cardiac imaging, mean ± SD**						
LVEF (%)	36 ± 9.5	✓		✓		
LVDed (mm)	58.8 ± 9.2					
LVDes (mm)	45.5 ± 9.7					
LVVed (mL)	152.7 ± 63.9					
LVVes (mL)	101.1 ± 51.6					
LAes (mm)	45.3 ± 7.2					
E/e'	12 ± 9.2					
E/A	1 ± 0.8					
Deceleration time (ms)	226 ± 80					
**Comorbidities, n (%)**						
Diabetes	82 (29)		✓	✓		
Hypertension	224 (80)					
Coronary artery disease	200 (71)	✓				
Smokers	125 (45)		✓	✓		
Hyperlipidemia	162 (58)					
COPD	9 (3)			✓		
**Medication, n (%)**						
ACE inhibitor	247 (88)	✓		✓		
Allopurinol	0 (0)	✓				
ARB	115 (41)	✓			✓	
Beta blocker	203 (73)	✓		✓		
Diuretics	219 (78)				✓	
Diuretic dose mg/kg per day	0.32 ± 0.31	✓				
Glycoside	59 (21)					
Aspirin	216 (77)					
Nitrate	146 (52)					
Antiarrhythmic agent	42 (15)					
Statin	114 (41)	✓			✓	

ACE, angiotensin-converting enzyme; ARB, angiotensin receptor blocker; BCN Bio-HF, Barcelona Bio-Heart Failure Risk Calculator; CLP, Cardiac Lipid Panel Risk Score; COPD, chronic obstructive pulmonary disease; E/A, ratio of the early (E) to late (A) ventricular filling velocities; E/e', ratio between early mitral inflow velocity and mitral annular early diastolic velocity; FRS, Framingham Risk Score; LAes, left atrial end systole; LDL, low-density lipoprotein; NYHA, New York Heart Association; HDL, high-density lipoprotein; LVDed, left ventricular diameter end diastole; LVDes, left ventricular diameter end systole; LVVed, left ventricular volume end diastole; LVEF, left ventricular ejection fraction; LVVes, left ventricular volume end systole; mg/kg, milligrams per kilograms; MAGGIC, Meta-analysis Global Group in Chronic Heart Failure; NTpro-BNP, N-terminal pro–B-type natriuretic; PC, phosphatidylcholine; SHFM, Seattle Heart Failure Model; SM, sphingomyelin; TAG, triacylglycerol.

*Imputed using the median, 31%, of the normal range 20–40%.

^†^Median (Interquartile Range).

All variables were available for each score except for the lymphocytes (%) variable in the SHFM score, which was imputed as previously described. The SHFM model had the highest number of variables (n = 17), followed by MAGGIC (n = 13), BCN Bio-HF (n = 12), FRS (n = 7), and CLP (n = 4). There were 13 overlapping variables which were included in at least 2 scores. The SHFM score included the most medication (n = 6) and laboratory (n = 5) variables, BCN Bio-HF is the only model with biomarker data (NT-proBNP) while MAGGIC included the most clinical (n = 7) and demographic variables (n = 3).

**Figure 1:**
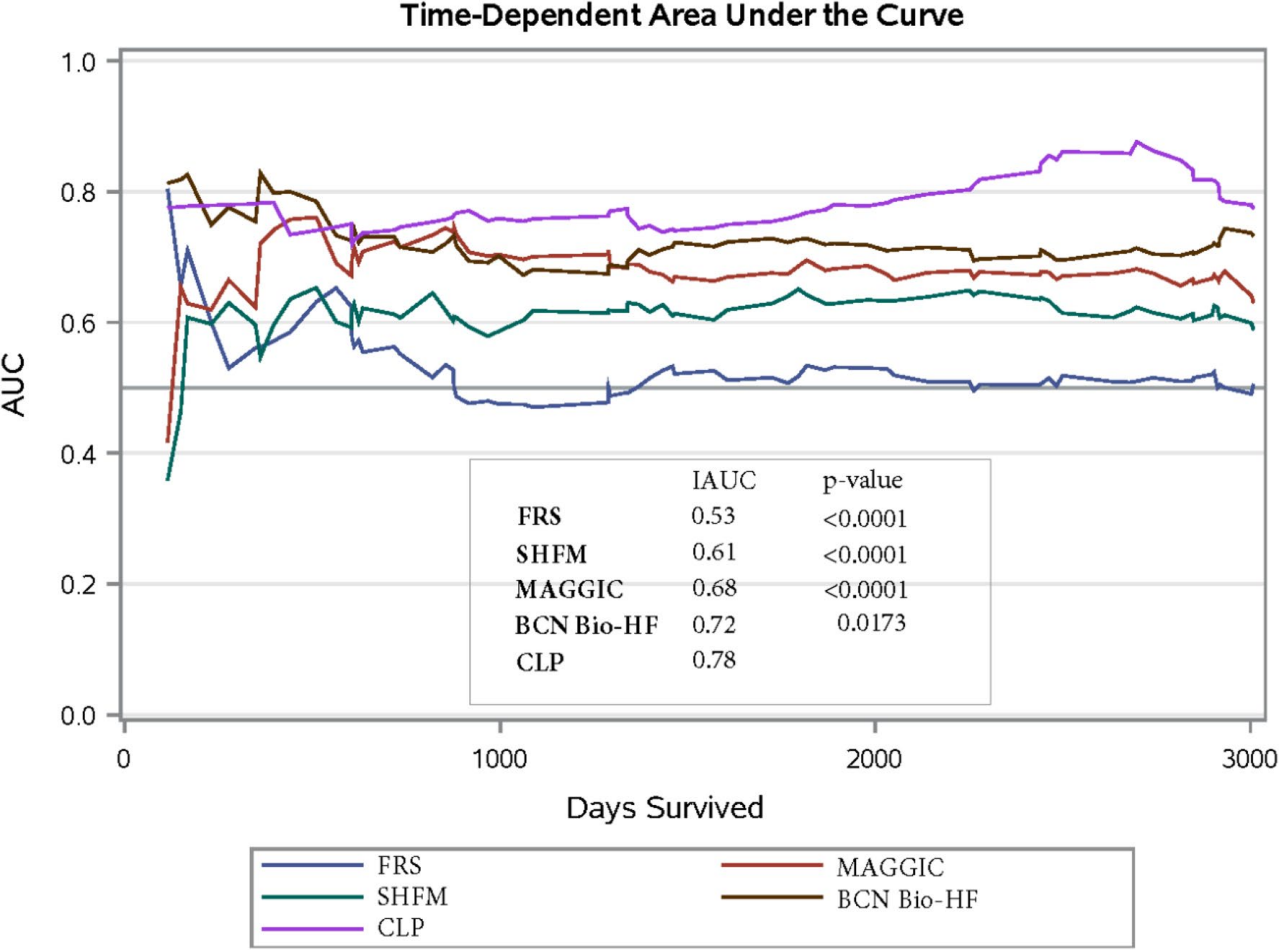
Discrimination performance for each prognostic score for 10-year cardiovascular mortality. Integrated area under the curve (IAUC) for: SHFM (Seattle Heart Failure Model), FRS (Framingham Risk Score), and MAGGIC (Meta-analysis Global Group in Chronic Heart Failure), BCN Bio-HF (Barcelona Bio-Heart Failure Risk Calculator), and Cardiac Lipid Panel Risk Score (CLP). Total subjects, n = 280; total events, n = 95. *p* values were calculated from the differences in concordance statistic in comparison to the CLP score.

Table 2 shows the univariate Cox Regression results**.** The CLP (HR = 2.38, *p* < 0.001), SHFM (HR = 2.01, *p* = 0.002, MAGGIC (HR = 1.10, *p* < 0.001), and BCN Bio-HF (HR = 1.09, *p* = 0.0393) scores were significantly associated with the outcome while FRS was not. The hazard ratios for the secondary endpoint of 3-year all-cause mortality are shown in Supplemental Table 1. All scores had a higher HR than the primary outcome except for CLP and FRS. Figure 1 shows the AUC change over time (IAUC) for the 5 prognostic scores with the comparison of Uno’s concordance statistics for hypothesis testing. The IAUC was 0.53, 0.61, 0.68, 0.72, and 0.78 for FRS, SHFM, MAGGIC, BCN Bio-HF, and CLP, respectively. Harrell’s c statistics at 10 year follow up show similar results (Supplemental Table 2). The four traditional scores were all significantly different (*p* < 0.05) from the CLP score according to the difference in concordance statistic (Supplemental Table 3). The incremental value of the CLP to NT-proBNP is shown in Supplemental Figure 1, the NT-proBNP only IAUC was 0.71 while the CLP score (which incorporates the CLP biomarkers plus NT-proBNP) was 0.78 (*p* = 0.004). Discrimination analysis of the secondary outcome of 3-year all-cause mortality showed the CLP IAUC lowered to 0.76, and only CLP vs FRS remained significantly different (Supplemental Figure 2). The models showed adequate calibration except for FRS (calibration curve slope = 0.894) (Supplemental Figure 3).

**Table 2:** Prognostic scores and univariate hazard ratios for cardiovascular mortality.

Score	HR (95% CI)	*p* value
SHFM	1.89 (1.29–2.807)	0.0017
FRS	1.02 (0.97–1.07)	0.5291
MAGGIC	1.10 (1.05–1.14)	< .0001
BCN Bio-HF	1.09 (1.00–1.84)	0.0393
CLP	2.38 (1.95–2.92)	< .0001

Unadjusted Cox proportional hazard models of 10-year outcome for cardiovascular mortality. Total subjects, n = 280. Total events, n = 95. SHFM (Seattle Heart Failure Model), FRS (Framingham Risk Score), and MAGGIC (Meta-analysis Global Group in Chronic Heart Failure), BCN Bio-HF (Barcelona Bio-Heart Failure Risk Calculator), and Cardiac Lipid Panel Risk Score (CLP).

Competing event analysis showed the SHFM, MAGGIC, and the CLP models remained significantly associated with cardiovascular death, and all scores showed less association to non-cardiovascular death (Supplemental Table 4). The CIF curve, which accounted for non-cardiovascular mortality as a competing event, showed higher cumulative incidence of cardiovascular mortality with higher CLP scores (Supplemental Figure 4).

**Figure 2:**
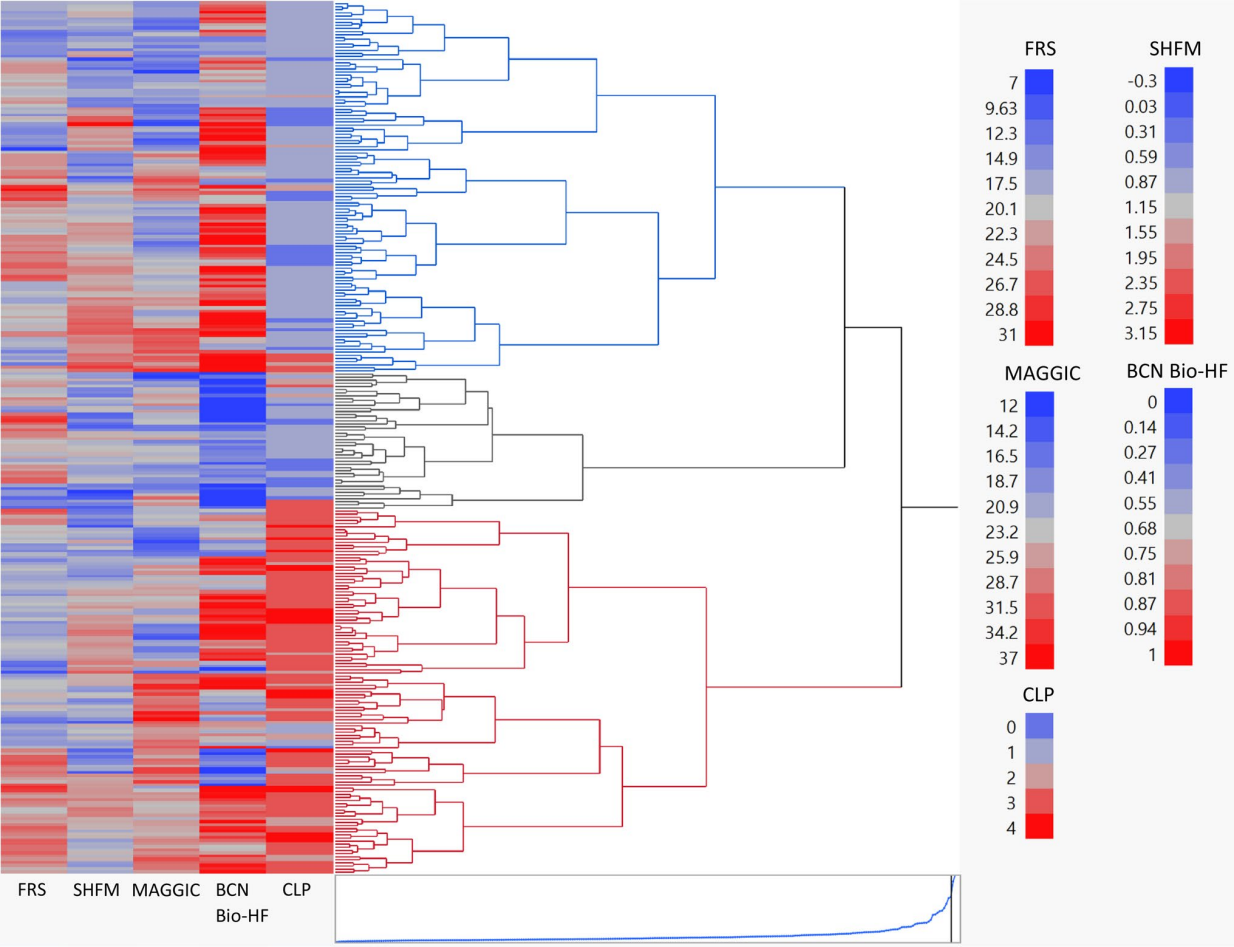
Hierarchal cluster dendrogram of three risk clusters. Assignment of patients into risk clusters based on the prognostic scores. The clustering process can be viewed by reading the dendrogram from left to right. Each step consists of combining the two closest clusters into a single cluster. The joining of clusters is indicated by horizontal lines that are connected by vertical lines. The horizontal position of the vertical line represents the distance between the two clusters that are most recently joined to form the specified number of clusters. The prognostic scores used for clustering were: SHFM (Seattle Heart Failure Model), FRS (Framingham Risk Score), and MAGGIC (Meta-analysis Global Group in Chronic Heart Failure), BCN Bio-HF (Barcelona Bio-Heart Failure Risk Calculator), and Cardiac Lipid Panel Risk Score (CLP). Each prognostic score was standardized to the same scale (mean = 0; SD = 1). Ward’s minimum variance method was used for clustering. Blue dendrogram indicates the cluster 1 (low risk), n = 119; Grey dendrogram indicates cluster 2 (moderate risk), n = 44, Red dendrogram indicates cluster 3 (high risk), n = 117; Total subjects, n = 280.

**Figure 3:**
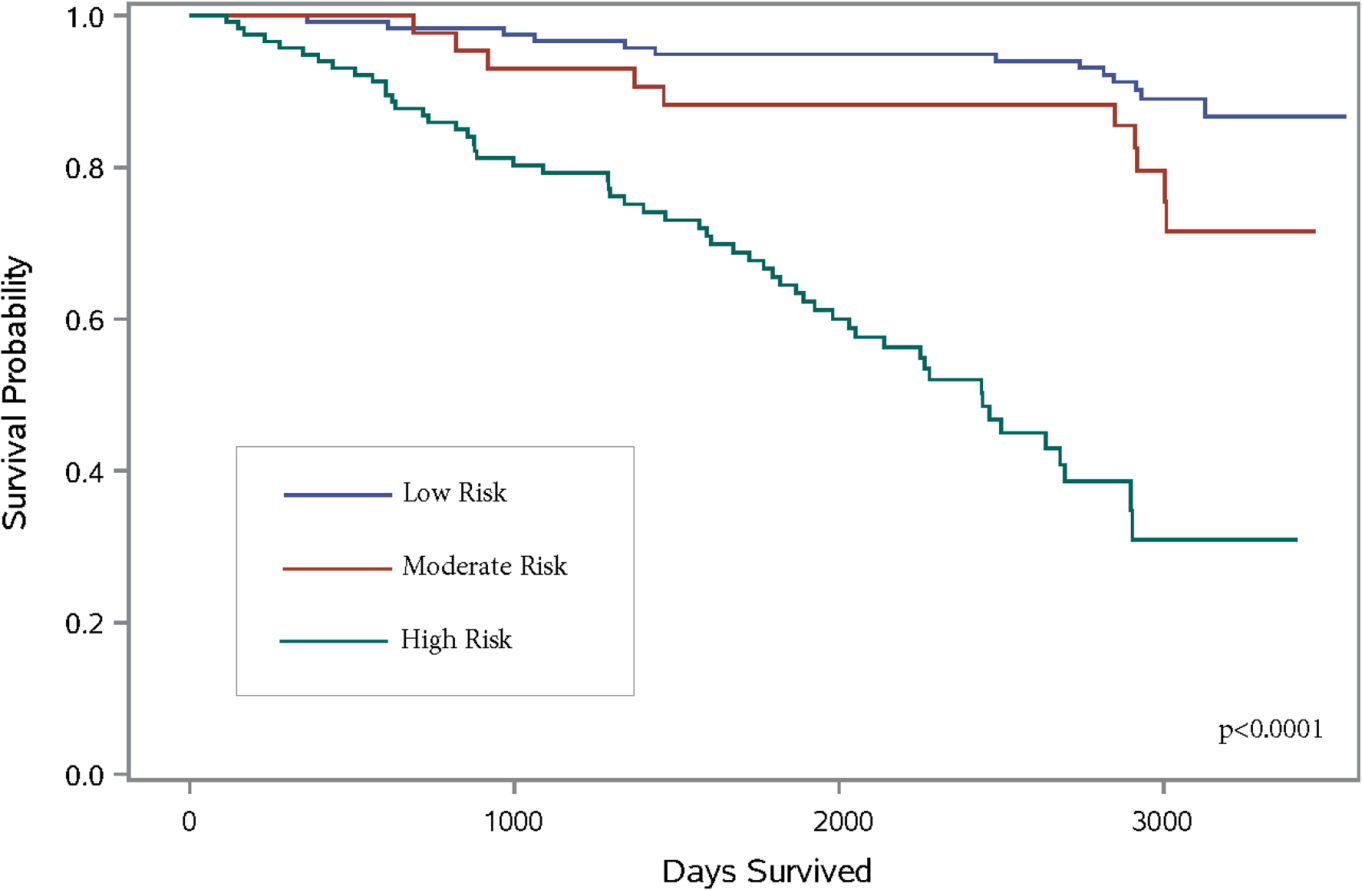
Kaplan Meier survival curves for 10-year cardiovascular mortality stratified by each risk cluster. The following scores were used to derive the risk clusters: SHFM (Seattle Heart Failure Model), FRS (Framingham Risk Score), MAGGIC (Meta-analysis Global Group in Chronic Heart Failure), BCN Bio-HF (Barcelona Bio-Heart Failure Risk Calculator), and Cardiac Lipid Panel Risk Score (CLP). Each prognostic score was standardized to the same scale (mean = 0; SD = 1). Total subjects, n = 280; total events, n = 95.

Figure 2 shows the hierarchical cluster dendrogram mapped to illustrate the assignment of patients into their respective clusters and the associated color map shows the range of each prognostic score and their distribution within each cluster. Hierarchical clustering grouped the patients in separate clusters accounting for the noise between smaller clusters. Each observation was treated as a unique cluster, and this method: (1) identified the two similar or close clusters, and (2) merged the two most similar clusters. Using this clustering technique, similar prognostic score data from participants were grouped together, such that the members in the same group were more similar to each other than the members in the other groups. We can infer from the cluster centres and cluster memberships that CLP risk score was better at grouping patients with respect to their cardiovascular mortality risk and associated clinical characteristics compared to the other four scores. The survival curves for each risk cluster are shown in Fig. 3. Rates of mortality were: low risk cluster (20%), moderate risk cluster (27%) and high-risk cluster (50%). Supplemental Figure 5 shows the constellation plot on a two-dimensional plane with nodes and links to describe relationship among component nodes. This plot is an alternate depiction of the dendrogram and illustrates the length between clusters and a balanced structure. Supplemental Figure 6 shows the scatterplot matrix of all 4 scores and clusters to illustrate the relationships between each prognostic score and risk cluster assignment.

Table 3 shows the cohort characteristics and the prognostic score distribution for each risk cluster. The three clusters were: low risk, n = 119; moderate risk, n = 44; high risk, n = 117. There were 11 out of the 50 cohort characteristics significantly different across the 3 clusters. In particular, patients in the highest risk cluster were older, with lower LVEF, higher NT-proBNP, and experienced a higher frequency of events. The SHFM, BCN Bio-HF, and CLP scoreswere significantly different across their respective risk clusters. Of the continuous risk scores (FRS, SHFM, MAGGIC, BCN Bio-HF), only SHFM and MAGGIC, had its highest mean score in the high-risk cluster. The categorical CLP score showed a skewed distribution of higher risk scores (3–4) in the moderate and high-risk clusters. In the high-risk cluster, the majority of subjects were scored with CLP scores of 3–4.

**Table 3:** Comparison of cohort characteristics and prognostic scores.

Characteristic	Cluster 1	Cluster 2	Cluster 3	*p* value
	Low risk	Moderate risk	High risk	
	n = 119	n = 44	n = 117	
Age (years), mean ± SD	71 ± 5.1	71 ± 4.2	73 ± 4.8	0.0058^a^
NYHA (II/III), n	83/36	35/9	71/46	0.1868^c^
Male, n (%)	91 (77)	29 (66)	86 (74)	0.3979^b^
Body mass index (kg/m^2^), mean ± SD	27.0 ± 3.0	27.0 ± 3.4	26.6 ± 3.9	0.4165^a^
Heart rate (bpm), mean ± SD	73.8 ± 26.7	72.0 ± 10.9	73.2 ± 12.6	0.9385^a^
Systolic blood pressure (mm Hg), mean ± SD	136 ± 17.7	137 ± 25.7	131 ± 16.9	0.0642^a^
Diastolic blood pressure (mm Hg), mean ± SD	82.9 ± 10.9	78.7 ± 13.0	79.5 ± 10.3	0.0485^a^
Years since first diagnosis of CHF	5.3 ± 5.9	5.0 ± 4.5	5.3 ± 5.7	0.9737^a^
Cardiac Death, n (%)	24 (20)	12 (27)	59 (50)	< 0.0001^b^
**Laboratory, mean ± SD**				
Serum creatinine (μmol/l)	103 ± 26.7	104 ± 26.7	113 ± 28.7	0.0034^a^
Hemoglobin (g/dL)	13.6 ± 1.4	13.4 ± 1.1	13.4 ± 1.7	0.1927^a^
Sodium (mmol/L)	142 ± 3.2	141 ± 3.6	141 ± 3.2	0.6576^a^
Uric acid (μmol/L)	342 ± 103	324 ± 108	378 ± 147	0.1084^a^
Total Cholesterol (mmol/L)	5.1 ± 1.6	5.4 ± 1.4	5.0 ± 1.6	0.2022^a^
HDL cholesterol (mmol/L)	1.2 ± 0.4	1.2 ± 0.4	1.2 ± 0.5	0.6042^a^
LDL cholesterol (mmol/L)	3.5 ± 1.4	3.5 ± 1.1	3.3 ± 1.3	0.4337^a^
Triglycerides (mmol/L)	1.7 ± 0.9	1.7 ± 0.9	1.8 ± 1.0	0.5816^a^
Lymphocytes (%)*	31	31	31	N/A
NT-proBNP (pg/mL)	506.0 (236–1461)^†^	860 (369–1883)^†^	1094 (450–2059)^†^	0.0015^a^
PC 16:0/18:2 (µg/dl)	36,830 (33,035–40,460)^†^	35,300 (30,538–39,370) ^†^	37,275 (31,688–39,850) ^†^	0.3678 ^a^
TAG 18:1/18:0/18:0 (µg/dl)	131 (91–253)^†^	107 (74–264) ^†^	103 (72–253) ^†^	0.5739 ^a^
SM d18:1/23:1, SM d18:2/23:0, SM d17:1/24:1 (µg/dl)	1433 (1181–1613)^†^	1378 (1156–1688) ^†^	1296 (1071–1529) ^†^	0.1598^a^
**Cardiac imaging, mean ± SD**				
LVEF (%)	37.7 ± 9.6	36.7 ± 8.2	34.0 ± 9.5	0.0046^a^
LVDed (mm)	57.8 ± 9.2	59.6 ± 8.1	59.5 ± 9.5	0.2684^a^
LVDes (mm)	44.3 ± 9.4	45.6 ± 9.0	46.7 ± 10.1	0.1422^a^
LVVed (mL)	147 ± 57.5	165 ± 77	154 ± 64.6	0.4697^a^
LVVes (mL)	95.9 ± 47	109 ± 60.7	104 ± 52.2	0.4449^a^
LAes (mm)	44.6 ± 6.9	45.3 ± 6.5	45.9 ± 7.7	0.3705^a^
E/e'	11.3 ± 8.8	10.4 ± 9.1	13.0 ± 9.5	0.0202^a^
E/A	1.0 ± 0.6	1.0 ± 0.8	1.2 ± 0.9	0.2407^a^
Deceleration time (ms)	233 ± 84.8	229 ± 80.8	219 ± 72.8	0.4823^a^
**Comorbidities, n (%)**				
Diabetes	29 (24)	11 (25)	42 (36)	0.1195^b^
Hypertension	95 (80)	39 (87)	90 (77)	0.2534^b^
Coronary artery disease	80 (67)	30 (68)	90 (77)	0.2245^b^
Smokers	55 (46)	20 (46)	50 (43)	0.8592^b^
Hyperlipidemia	67(56)	26 (61)	69 (59)	0.8636^b^
COPD	2 (2)	2 (5)	5 (4)	0.2143^b^
**Medication, n (%)**				
ACE inhibitor	104 (87)	36 (82)	107 (92)	0.2244^b^
Allopurinol	0 (0)	0 (0)	0 (0)	N/A
ARB	39 (33)	16 (36)	60 (51)	0.0121^b^
Beta blocker	87 (73)	36 (82)	80 (68)	0.2303^b^
Diuretics	53 (45)	23 (52)	69 (59)	0.0850^b^
Diuretic dose mg/kg, mean ± SD	0.28 ± 0.25	0.33 ± 0.35	0.36 ± 0.34	0.3410^a^
Glycoside	26 (22)	8 (18)	25 (21)	0.8736^b^
Aspirin	92 (77)	33 (75)	91 (78)	0.9309^b^
Nitrate	55 (46)	28 (64)	63 (54)	0.1262^b^
Antiarrhythmic agent	16 (13)	6 (14)	20 (17)	0.7075^b^
Statin	51 (43)	18 (41)	45 (39)	0.7894^b^
**Prognostic scores**				
SHFM, Mean (SD)	1.1 ± 0.54	0.9 ± 0.6	1.32 ± 0.5	< .0001^a^
FRS, Mean (SD)	20.1 ± 4.1	20.6 ± 4.5	20.1 ± 4.0	0.6033^a^
MAGGIC, Mean (SD)	22.1 ± 5.0	21.4 ± 3.8	22.7 ± 5.0	0.2092^a^
BCN Bio-HF, Mean (SD)	0.83 ± 0.17	0.34 ± 0.13	0.76 ± 0.2	< .0001^a^
**CLP, n (%)**				< .0001^b^
0	6 (6)	1 (3)	0 (0)	
1	83 (85)	24 (75)	4 (4)	
2	4 (4)	3 (9)	13 (12)	
3	5 (5)	4 (13)	77 (68)	
4	0 (0)	0 (0)	19 (17)	

Cohort characteristics and prognostic score distribution across risk clusters. The prognostic scores used for clustering were: SHFM (Seattle Heart Failure Model), FRS (Framingham Risk Score), and MAGGIC (Meta-analysis Global Group in Chronic Heart Failure), BCN Bio-HF (Barcelona Bio-Heart Failure Risk Calculator), and Cardiac Lipid Panel Risk Score (CLP). Each prognostic score was standardized to the same scale (mean = 0; SD = 1). Ward’s minimum variance method was used for clustering.

ACE, angiotensin-converting enzyme; ARB, angiotensin receptor blocker; TAG, triacylglycerol 18:1/18:0/18:0; COPD, chronic obstructive pulmonary disease; E/A, ratio of the early (E) to late (A) ventricular filling velocities; E/e', ratio between early mitral inflow velocity and mitral annular early diastolic velocity; LAes, left atrial end systole; LDL, low-density lipoprotein; NYHA, New York Heart Association; HDL, high-density lipoprotein; LVDed, left ventricular diameter end diastole; LVDes, left ventricular diameter end systole; LVVed, left ventricular volume end diastole; LVEF, left ventricular ejection fraction; LVVes, left ventricular volume end systole; mg/kg, milligrams per kilograms; NTpro-BNP, N-terminal pro–B-type natriuretic; PC, phosphatidylcholine; SM, sphingomyelin.

^a^Wilcoxon rank sum test, ^b^Pearson’s chi-square test, ^c^Mantel-Haenszel chi-square.

*Imputed using the median, 31%, of the normal range 20–40%.

^†^Median (Interquartile Range).

The correlation of the CLP biomarkers TAG, PC, and SM were most correlated with the clinical characteristics: triglycerides (r = 0.531, *p* < 0.001), total cholesterol (r = 0.431, *p* < 0.001), and LDL (r = 0.502, *p* < 0.001), respectively (Supplemental Figure 7).

## Discussion

We found that a risk score based on a novel panel of three metabolite-based biomarkers plus NT-proBNP outperformed commonly used traditional prognostic models for predicting cardiovascular mortality in elderly ambulatory CHF patients. We first measured the association of each risk score with the outcome, followed by discrimination analysis, then cluster analysis, and finally correlation analysis of the individual CLP biomarkers with the clinical characteristics. In our study cohort, CLP score, showed the best discrimination compared to the other 4 scores. This indicates that the biomarker information included in the CLP score could more precisely classify high risk CHF patients than the information included in the 4 other risk scores. On the other hand, the biomarker information from the CLP is not as easily attainable and no convenient calculator exists yet, as these findings should first be validated in larger cohorts. Additionally, none of the other scores were originally developed for 10-year cardiovascular mortality. To the best of our knowledge there is no score specific for predicting 10-year risk of cardiovascular death, but it is not uncommon to use the scores such as FRS to predict different outcomes in similar studies^31–33^. Nevertheless, the other risk scores may be improved with the addition of common biomarkers in their score calculation. For instance, NT-proBNP is a well-established biomarker that is known to be associated with ventricular wall stress^34^ and is considered the gold-standard biomarker in CHF diagnosis and prognosis^35^. Only BCN Bio-HF contained NT-proBNP and it was the next best performing prognostic score after the CLP.

We performed cluster analysis to assess how well the risk scores could partition subjects into different risk groups, blinded to the study outcome. A strength of this approach is that clusters could define relevant groups of patients and could mitigate the problems of multicollinearity while determining if the predictive variables are useful in separating these groups. In our study, patients within each cluster varied along measures of age, laboratory parameters, days survived, as well as the prognostic scores. When comparing the score distributions across the three risk clusters, the CLP score showed a more homogenous grouping of patients according to their risk score stratification while the other scores showed a more heterogenous distribution across risk clusters. Several prior studies have used similar clustering methods to identify clinically relevant patient subgroups for CHF^36,37^, but we are not aware of previous studies using clustering methods to compare a novel biomarker score to other conventional prognostic scores for CHF.

The combination of the CLP’s metabolomic features with NT-proBNP into a risk score may help overcome limitations of using only traditional clinical risk factors. Furthermore, application of a single biomarker such as NT-proBNP for outcome prediction is limited by insufficient specificity (low predictive value or high false positive rate)^38,39^. Recently, it was reported that the CLP added incremental prognostic value to NT-proBNP in predicting 4-year cardiovascular mortality^30^. We used the same method to calculate the CLP score for this study, and we also confirmed that the CLP provided similar incremental value to NT-proBNP alone as previously found in the 4-year study^30^.Using an aggregate score rather than individual biomarkers for risk prediction can help more precisely stratify risk. A recent meta-analysis of 18 metabolomic prognostic biomarker studies for CVD found those which incorporated a selection of metabolites into a score (n = 5 studies) had the best prognostic performance rather than using the individual biomarker values^16^. Another systematic review^20^ reported 6 studies^21–26^ developed a metabolite-based score to predict CVD risk with each score composed between 4 and 16 biomarkers.

We have briefly mentioned the components of the CLP in the introduction section, in addition to improving risk prediction, developing a biomarker-based risk score could also improve our understanding of the pathophysiology and biological mechanisms involved in CHF. In the following paragraphs we would like to highlight those mechanisms based on previous research. The CLP metabolites can be grouped into three different lipid subclasses, sphingomyelin (SM) phosphatidylcholine (PC), and triglycerides (TAG), which have previously been found to be associated with cardiomyocyte stress/apoptosis^40^, intestinal microbial metabolism/inflammation^19^, and coronary artery disease^41^, respectively. Sphingomyelins are localized in cell membranes and lipoproteins, and their hydrolysis by sphingomyelinase leads to increased amounts of ceramide. Ceramide triggers the generation of reactive oxygen species (ROS) involved in the modulation of cell proliferation and apoptosis, neutrophil adhesion to the vessel wall, and vascular tone. Dysfunctional sphingomyelin and ceramide metabolism may lead to or aggravate cardiovascular diseases^42^. Lemaitre et al.^43^ reported that lipid species such as Cer-16 and SM-16 were associated with increased risk of heart failure. Sigruener et al.^44^ reported that the detection of sphingomyelin species SM 16∶0, 16∶1, 24∶1 and 24∶2 was increasingly associated with mortality in Ludwigshafen Risk and Cardiovascular Health (LURIC) study. The CLP biomarker panel consists of the sum of three monosaturated fatty acid carrying SM species: SM d18:1/23:1, SM d18:2/23:0, SM d17:1/24:1.

PC is the most abundant lipid in the human body and is subjected to chemical events like lipid peroxidation and ROS formation^45^. Myocardium suffers heavily from lipid peroxidation related injury^46^. PC carrying polyunsaturated fatty acids such as PC (16:0/18:2) which is a component of the CLP panel, have increased risk for lipid peroxidation^47^. Oxidative stress increases the formation of electrophilic aldehydes from native phospholipids leading to formation of adducts with tissue or plasma proteins thereby aggravating the pathophysiology of cardiovascular diseases^48^. Previous studies have shown that lipid peroxidation and ROS generation are associated with cardiac damage and raises mortality. Higher consumption of PC was found to increase the risk of organ injury and cardiovascular mortality^49^. Natural antioxidants like α-tocopherol have shown to reduce such oxidative stress and resulting inflammation thereby preventing the progression of cardiac injury^50^.

The molecules of TG are involved in the regulation of insulin-signaling pathways through the activation of several serine/threonine kinases, which suppress insulin receptors, thus inducing peripheral insulin resistance. Previous studies have shown that insulin resistance leads to inflammation and atherosclerosis^51^. Although the relationship between total triglycerides and insulin resistance and CVD risk are well established^52^, the relationships between individual serum TGs and insulin resistance is not well-established. Studies of individual TGs may help better characterize insulin resistance and CVD better than total triglycerides. For instance, it was previously found that saturated TG 16:0 fatty acid was positively associated with fasting serum insulin concentrations and that of essential 18:3 n-6 fatty acid was negatively associated^53^. Another study on individual TGs revealed that serum TG molecules containing saturated and monounsaturated fatty acids, such as TG(16:0/16:0/18:1) and TG(16:0/18:1/18:0), correlated positively, whereas those containing essential fatty acids, such as TG(18:1/18:2/18:2), negatively with features of insulin resistance^54^. The CLP consists of the saturated and monosaturated fatty acid carrying TAG 18:1/18:0/18:0.

These findings indicate that metabolomic studies may help gain a deeper understanding of the molecular mechanisms of CVD. Therefore, more detailed metabolomic analysis would hopefully lead to the discovery or further development of sensitive and specific lipid-based markers for cardiovascular risk.

## Study limitations

These proof-of-concept findings should be interpreted as hypothesis generating to be used as a reference for validation studies on larger cohorts in the future. The homogeneity of this cohort, elderly patients with stable CHF, may have had an impact on the performance of the prognostic scores. Due to the inclusion and exclusion criteria of the CIBIS-ELD trial, these results may not have good external validity, and more research would be needed to validate the results. Performance and comparison of the risk scores may be affected by the fact that the models were designed using different endpoint definitions and cohorts. Risk categories that are clinically relevant for one model’s definition may not apply to a different model. The MAGGIC score estimates risk of all-cause mortality at one and at three years, the SHFM up to five years, and the BCN Bio-HF at one, two, and three years, and the FRS estimates risk of first CVD event, none of which were developed for the primary outcome of this study of 10-year cardiovascular mortality. The SHFM score may have been affected by the imputation of lymphocytes % as well as the lack of patients taking allopurinol. The BCN-bio HF score was updated in 2018^55^ which could provide better predictive value than the 2014 version used in this study. We were limited by the availability of the data for the 2018 version of the BCN-bio HF score, since it required more parameters such as ARNi medication and number of HF hospitalizations in the previous year. The FRS was originally developed for coronary artery disease and not CHF, which may explain its poor performance on this cohort. The CLP biomarker kit was developed for routine use in the clinic; however, it is still a research biomarker panel pending regulatory approval and must be sent to a lab equipped with MS technology. Our findings are limited to this population of elderly CHF patients and future validation studies should be performed to include a more heterogenous cohort such as younger, more women, and early/ asymptomatic patients. Other common biomarkers such as ST2, hs-CRP, and troponins should be compared to the CLP as they are more readily available and do not require samples be sent to a specialized lab. The CLP panel was originally developed as a diagnostic and early detection biomarker for HFrEF, and clinicians and researchers should be cautious when using it as a prognostic tool, as these are still preliminary findings.

## Conclusion

In a cohort of ambulatory CHF patients, we have shown that the prognostic scores included in this study were useful in stratifying patients into risk clusters. Our findings demonstrate that the CLP risk score comprising a panel of 3 novel metabolomic biomarkers and NT-proBNP, could improve the prediction of cardiovascular mortality over traditional prognostic scores. In the future, a broader array of biomarkers should be integrated into a more comprehensive risk score that may improve discrimination potential and risk stratification and the CLP offers a promise. The CLP score is a step in the direction of providing a more precise decision support tool to assist clinicians and patients in managing their CHF treatment.

## Methods

### Study population

This study used a sub-cohort randomly selected from the Cardiac Insufficiency Bisoprolol Study in Elderly (CIBIS-ELD) trial, a multi-center, randomized, double-blind trial with ≥ 65-year-old patients being treated for CHF. The original study design and results of the CIBIS-ELD trial have been published previously^56,57^. Briefly, patients with CHF were randomized in a 1:1 fashion to receive two different beta-blockers, either bisoprolol or carvedilol, and up titrated every fortnight for 12 weeks and then followed at 10 years. From this source cohort (n = 883), there were n = 589 with available blood samples. Patients were randomly selected and included in the analysis only if they passed quality control^58,59^ resulting in a final set of 280 cases. The ethics committees of all participating centers approved the study protocol, and informed consent was signed by all participants prior to study participation. The ethics committees include: Germany: Ethikkommission der Charité on the 13th June 2007 (Amendment 5) (ref: 125/2004), Serbia: Ethics board of the University Hospital on the 31st March 2006 (ref: 6108/18), Slovenia: The national medical ethics committee on the 2nd July 2007 (ref: KME 188/06/07). The investigation conformed to the principles outlined in the Declaration of Helsinki^60^.

### Biomarker measurements

Targeted metabolite profiling of the serum samples which passed quality control was performed at a specialized metabolomics lab using a commercially available kit. The kit uses a protocol based on a 1-phase extraction of the blood samples followed by gas chromatography mass spectrometry (GC–MS) (Agilent 6890 GC coupled to an Agilent 5973 MS-System) and liquid chromatography tandem-mass spectrometry (LC–MS/MS) (Agilent 1100 HPLC-System coupled to an Applied Biosystems API4000 MS/MS-System) analysis as previously described^29^. The analytical protocol was designed for routine measurement in the clinical practice setting; however, it is currently only available in specialized labs equipped with MS technology. The samples were stored at − 80 °C and transferred on dry ice prior to analysis. The three CLP metabolomic features and NT-proBNP measurements, were generated at baseline, only for the previously mentioned samples (n = 280). NT-proBNP was a measured using commercially available assays (Elecsys, Roche Diagnostics).

### Calculating prognostic scores

Each prognostic score was calculated using the corresponding method proposed by the original authors (3–6). Only the scores which were developed in the follow-up time period, 2006–2016, were included in the analysis due to data availability. For calculating the SHFM score, % lymphocyte was missing, and the median (31%) of the normal range (20–40%) was imputed for all subjects. For calculating the BCN Bio-HF score, the model with clinical variables plus NT-proBNP was used since ST-2 and hs-cTnT were not available. The CLP risk score was calculated as the count of biomarkers above the Youden index cut-off^61^. The Youden’s index calculates each biomarker’s optimal cut-off from the Cox regression. There were 4 cut-off values, since four biomarkers are included in the score: three from the CLP and NT-proBNP. Based on the cut-off, a value of 1 or 0 was assigned if the biomarker value was above/below the cut-off value, or in the direction of greater risk, then all 4 values were summed to generate the final score for each subject. The score ranged from 0 to 4, higher scores indicating higher risk. The primary outcome, cardiovascular death, was defined as death by myocardial infarction, non-responding arrhythmia, asystole, chronic pump failure, or other cardiac cause and verified by a blinded committee of cardiologists.

### Statistical analysis

#### Power and sample Size

The sample size was adjusted for an anticipated event rate of 0.34. A Cox regression of the log hazard ratio on a covariate with a standard deviation of 1.5 based on a sample of 257 observations achieves 80% power at a 0.050 significance level to detect a regression coefficient equal to 0.2. Adjusting for an anticipated loss to follow up rate of 10%, the final sample size would be 283.

#### Discrimination analysis and calibration

Categorical variables were expressed as number (%) and continuous variables were expressed as mean (*SD*). The primary outcome was 10-year cardiovascular death, and the secondary outcome was 3 year all-cause death, since all scores except for FRS were developed for this outcome. Cox Regression was performed on each of the prognostic scores, and hazard ratios and 95% confidence intervals were calculated to assess their relationship with the outcome.

For the survival models, integrated area under the receiver operator curves (IAUC) and Harrell’s c statistic^62^ were calculated to assess the discrimination of each score in predicting the outcome. Hypothesis testing of the change in discrimination was performed by calculating the differences in concordance statistics^63^. The IAUC curves are computed as a weighted average of the AUC values at all the event times, with the weights as the jumps of the Kaplan–Meier estimate of the survivor function. Calibration (i.e., the agreement between observed outcomes and predictions) of all models was assessed graphically, with calibration plots.

Competing event and cause-specific analysis was performed for all models with non-cardiovascular mortality as the competing event. The cumulative incidence function (CIF) was calculated for the CLP which was stratified by low (CLP score 0–1), moderate (CLP score 2), and high (CLP score 3–4) to assess CIF. The discrimination analysis and competing event and cause-specific analysis were performed using SAS software version 9.4 of the SAS System for Windows (SAS Institute, Inc., Cary, North Carolina)^64^. Calibration was analyzed using Stata Statistical Software version 16^65^.

#### Cluster analysis

Hierarchical cluster analysis was performed using Ward’s minimum variance method to assess each prognostic score’s ability to separate cases into risk groups. The distance between two clusters is the ANOVA sum of squares between the clusters summed over all variables. Only the 5 risk scores used as the input variables for the cluster analysis to examine how well they classified patients into a low, moderate, and high-risk of cardiovascular mortality. Data was standardized (mean of 0 and SD of 1), to perform clustering. The clinical characteristics and scores were compared across risk clusters. Comparisons among continuous variables were performed using Wilcoxon rank sum test; and Pearson’s chi-square test (or Fisher’s exact test) or Mantel–Haenszel Chi-square test for categorical and ordinal data, respectively. Kaplan–Meier curves were used to compare the survival distribution across risk clusters. Survival time was calculated from baseline until cardiovascular death or censoring at 10 year follow up. Cluster analysis was performed using JMP pro software version 14^66^. Kaplan–Meier curves were generated using SAS software version 9.4 of the SAS System for Windows (SAS Institute, Inc., Cary, North Carolina)^64^.

#### Correlation analysis

To investigate potential relationships between the CLP biomarker values and common clinical parameters, Pearson’s correlation coefficients were calculated, significant at the 0.01 level (2-tailed). Correlation analysis was performed using R software version 3.6.1^67^.

## Supplementary Information

Supplementary Information 1.

Supplementary Information 2.

Supplementary Information 3.

Supplementary Information 4.

Supplementary Information 5.

Supplementary Information 6.

Supplementary Information 7.

Supplementary Information 8.

Supplementary Information 9.

Supplementary Information 10.

Supplementary Information 11.
