# Supplementary material for: Performance of a cardiac lipid panel compared to four prognostic scores in chronic heart failure
Source: Sci Rep. 2021 Apr 14;11:8164. doi: 10.1038/s41598-021-87776-w (PMC8046832; doi:10.1038/s41598-021-87776-w)
Supplement: Supplementary file 1 — Supplementary Information 1. [file 41598_2021_87776_MOESM1_ESM.docx]

**Supplemental Figure 1: Incremental value of the CLP compared to NT-proBNP only for 10-year cardiovascular mortality**

**
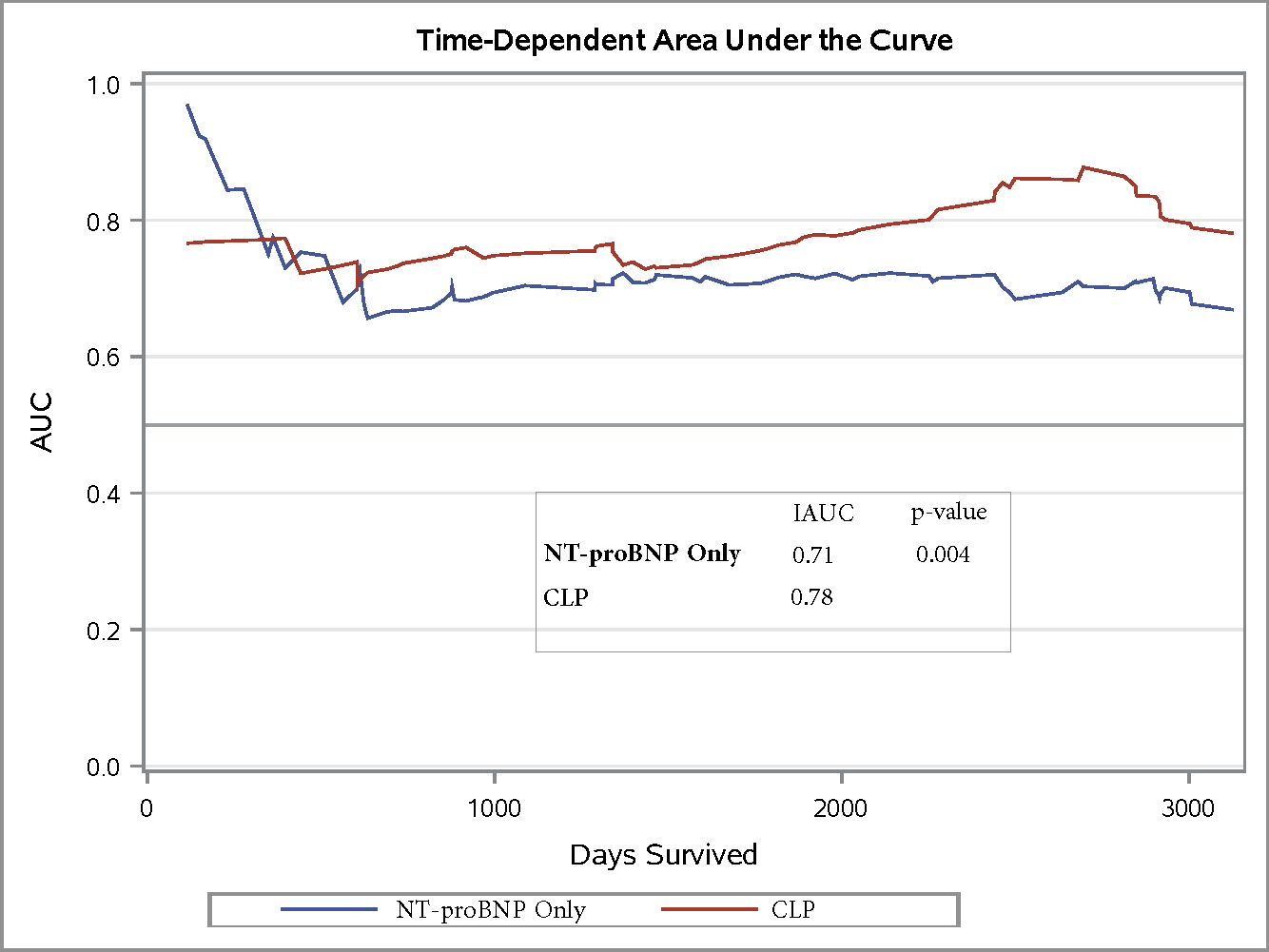
**

Caption: Integrated area under the curve (IAUC) for N-terminal pro-brain natriuretic peptide (NT-proBNP) and Cardic Lipid Panel score (CLP). P-values were calculated from the differences in concordance statistic. Total subjects, n=280; total events, n=95.
