# Supplementary material for: Performance of a cardiac lipid panel compared to four prognostic scores in chronic heart failure
Source: Sci Rep. 2021 Apr 14;11:8164. doi: 10.1038/s41598-021-87776-w (PMC8046832; doi:10.1038/s41598-021-87776-w)
Supplement: Supplementary file 2 — Supplementary Information 2. [file 41598_2021_87776_MOESM2_ESM.docx]

**Supplemental Figure 2: Discrimination Performance for each Prognostic Score for 3-year All-Cause Mortality**


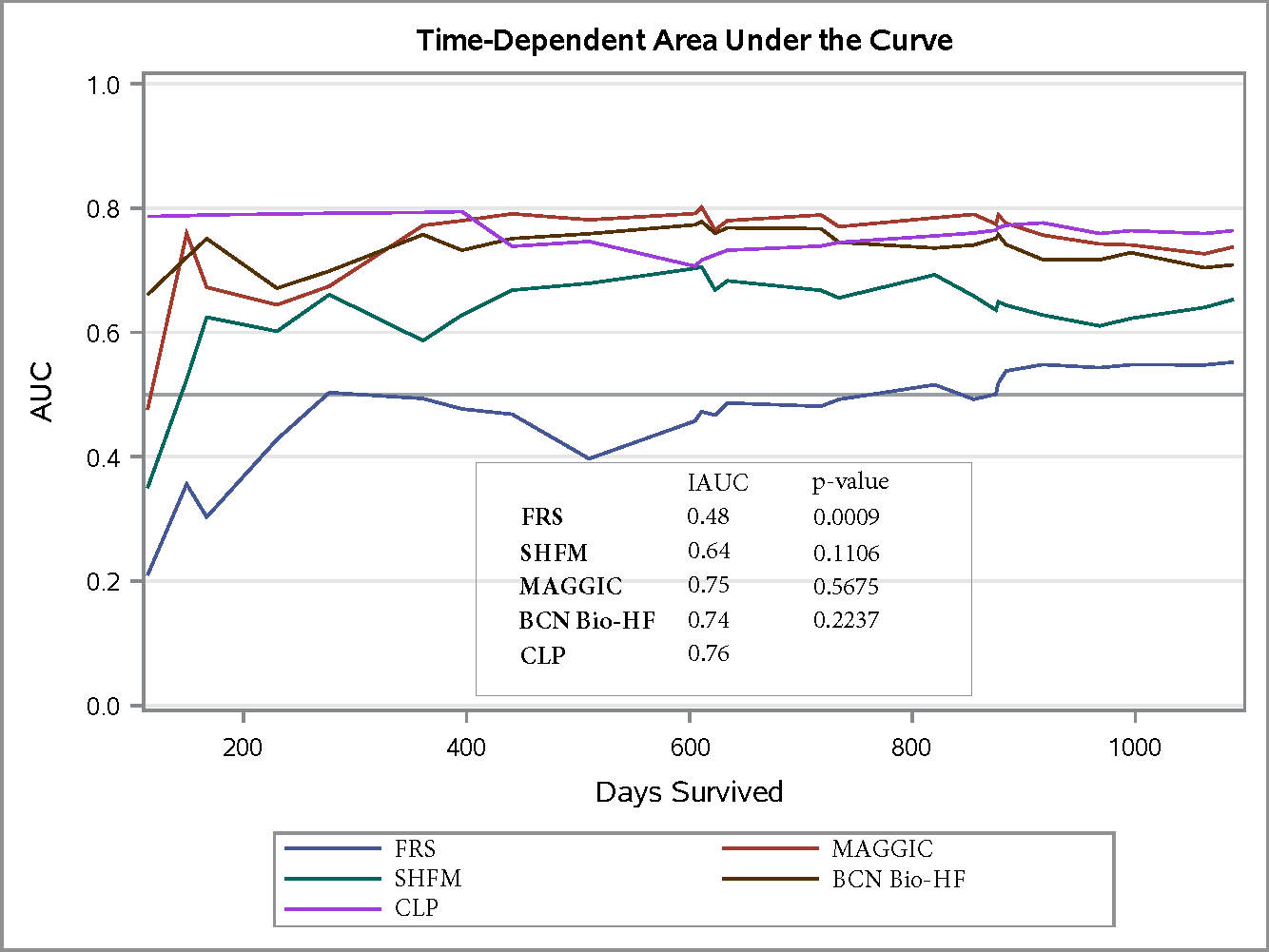


Caption: Integrated area under the curve (IAUC) for: SHFM (Seattle Heart Failure Model), FRS (Framingham Risk Score), and MAGGIC (Meta-analysis Global Group in Chronic Heart Failure), BCN Bio-HF (Barcelona Bio-Heart Failure Risk Calculator), and Cardiac Lipid Panel Risk Score (CLP). Total subjects, n=280; total events, n=51.

P-values were calculated from the differences in Uno’s concordance statistic in comparison to the CLP score.
