# Supplementary material for: Performance of a cardiac lipid panel compared to four prognostic scores in chronic heart failure
Source: Sci Rep. 2021 Apr 14;11:8164. doi: 10.1038/s41598-021-87776-w (PMC8046832; doi:10.1038/s41598-021-87776-w)
Supplement: Supplementary file 3 — Supplementary Information 3. [file 41598_2021_87776_MOESM3_ESM.docx]

**Supplemental Figure 3: Calibration plots of the five prognostic scores**

A B


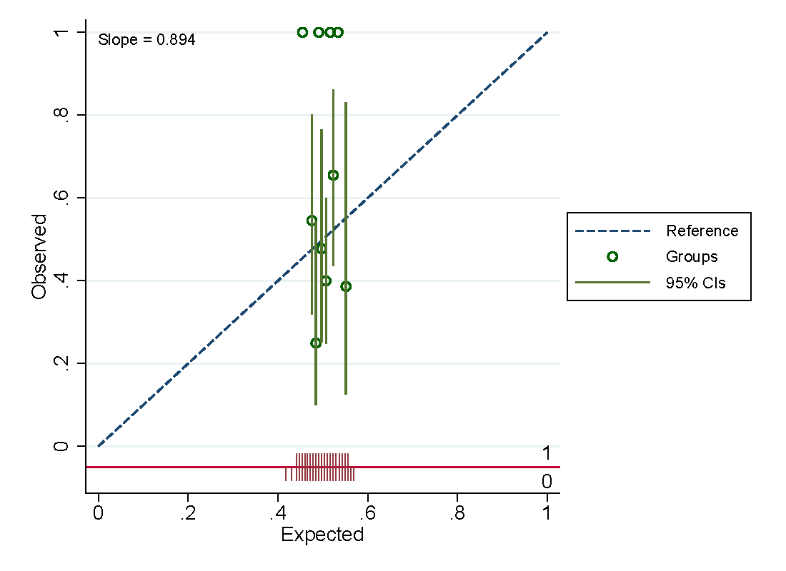

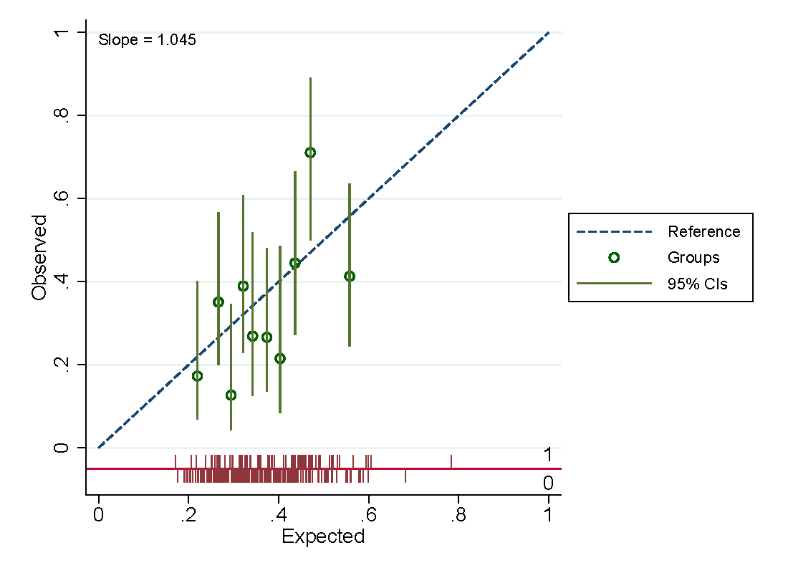


C D


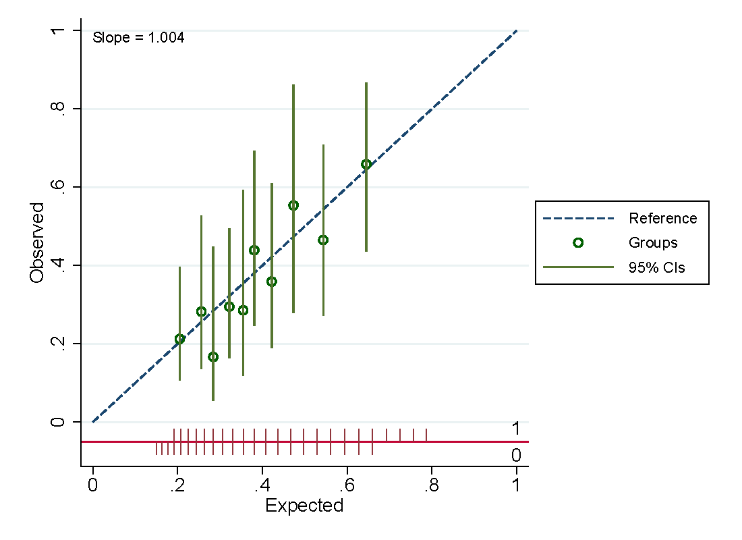

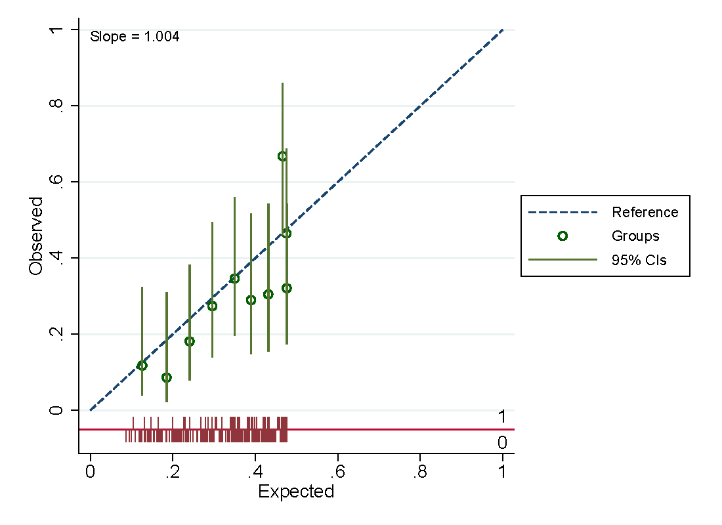


E


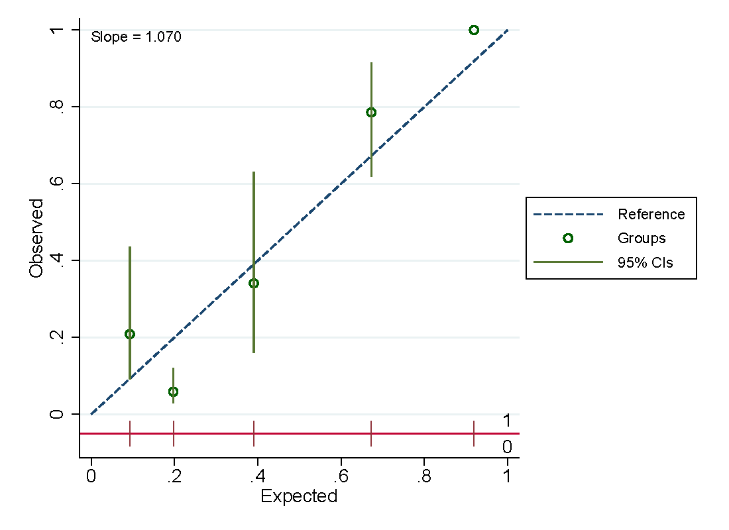


Caption:

A: FRS (Framingham Risk Score); B: SHFM (Seattle Heart Failure Model); C: MAGGIC (Meta-analysis Global Group in Chronic Heart Failure), D: BCN Bio-HF (Barcelona Bio-Heart Failure Risk Calculator); E: CLP (Cardiac Lipid Panel Risk Score). The dotted line represents perfect calibration. Scatter points are displayed with 95% confidence intervals of observed cardiovascular mortality. The slope of the observed vs predicted line is given. The number of bins were used to group patients average observed & expected probabilities. Total subject, n=280. Total events, n=95.
