# Supplementary material for: Performance of a cardiac lipid panel compared to four prognostic scores in chronic heart failure
Source: Sci Rep. 2021 Apr 14;11:8164. doi: 10.1038/s41598-021-87776-w (PMC8046832; doi:10.1038/s41598-021-87776-w)
Supplement: Supplementary file 4 — Supplementary Information 4. [file 41598_2021_87776_MOESM4_ESM.docx]

**Supplemental Figure 4: Cumulative incidence curves for the CLP competing events model**


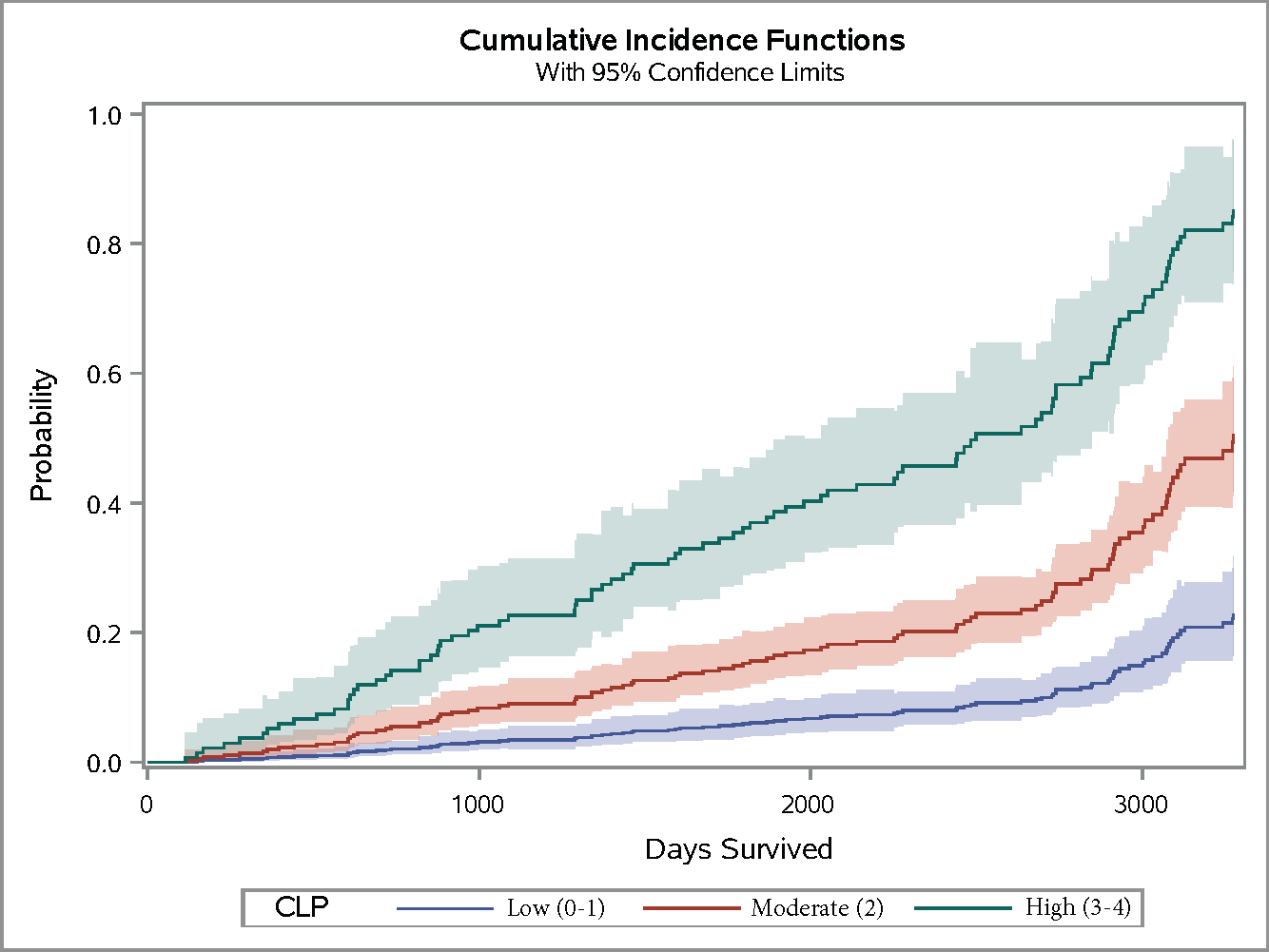


Caption: The event of interest is cardiovascular death, n=95. The competing event is non-cardiovascular death, n=51. Total subjects, n=280. CLP (Cardiac Lipid Panel); CLP scores were grouped 0-1 = Low; 2=Moderate; 3-4 = High. Color bands represent 95% confidence intervals
