# Supplementary material for: Performance of a cardiac lipid panel compared to four prognostic scores in chronic heart failure
Source: Sci Rep. 2021 Apr 14;11:8164. doi: 10.1038/s41598-021-87776-w (PMC8046832; doi:10.1038/s41598-021-87776-w)
Supplement: Supplementary file 5 — Supplementary Information 5. [file 41598_2021_87776_MOESM5_ESM.docx]

**Supplemental Figure 5: Constellation plot of the hierarchical cluster analysis**


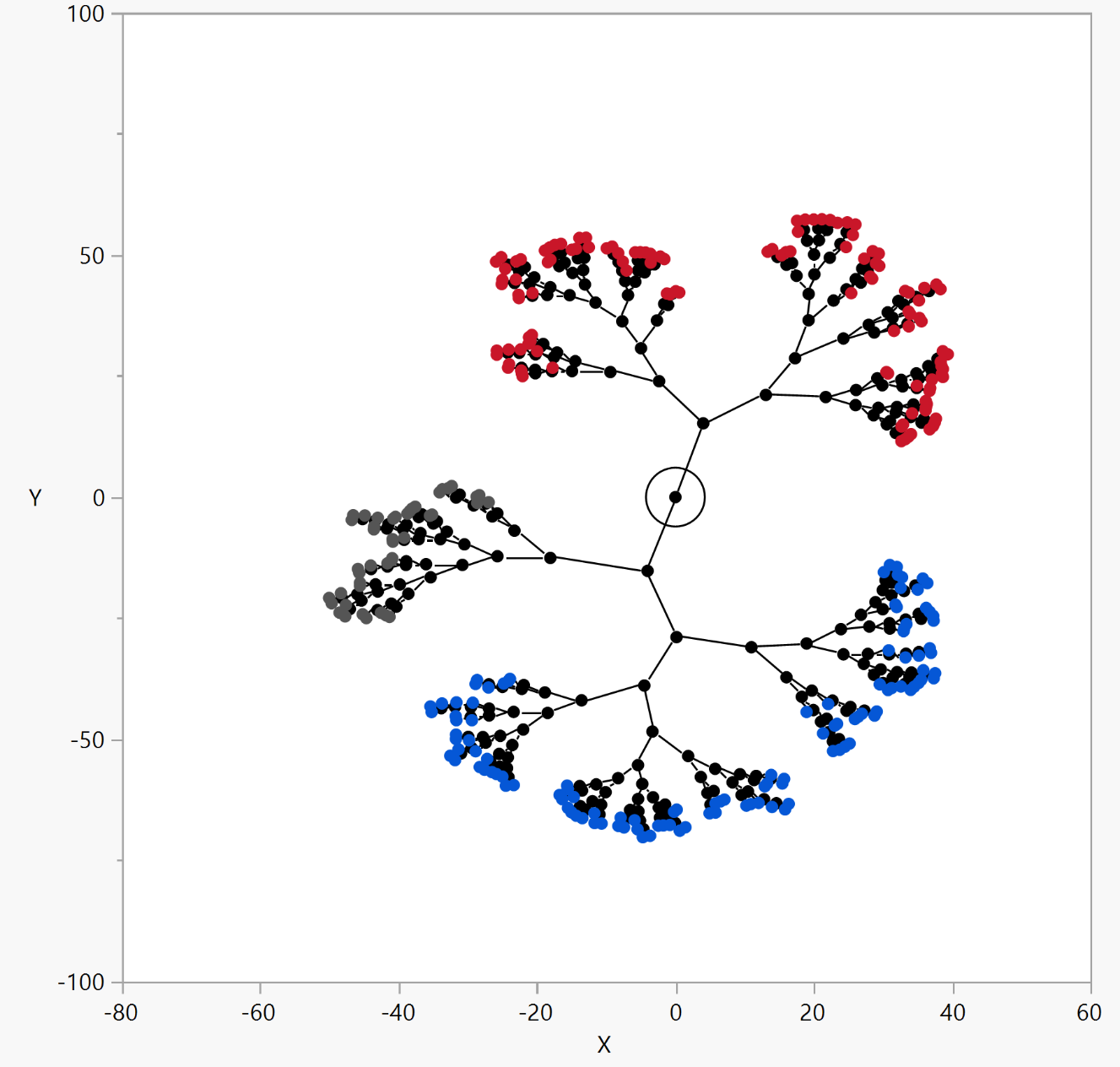


Caption:

Alternate depiction of the hierarchical cluster analysis dendrogram. Each endpoint represents a patient. The lengths of the lines represent the distance between clusters. The axis scaling, orientation of points, and angles of the lines are arbitrary.

Blue dots indicate cluster 1 (low risk), n=119; Grey dots indicate cluster 2 (moderate risk), n= 44; Red dots indicate cluster 3 (high risk), n= 117.
