# Supplementary material for: Performance of a cardiac lipid panel compared to four prognostic scores in chronic heart failure
Source: Sci Rep. 2021 Apr 14;11:8164. doi: 10.1038/s41598-021-87776-w (PMC8046832; doi:10.1038/s41598-021-87776-w)
Supplement: Supplementary file 6 — Supplementary Information 6. [file 41598_2021_87776_MOESM6_ESM.docx]

**Supplemental Figure 6: Scatterplot matrix of risk clusters for each prognostic risk score**


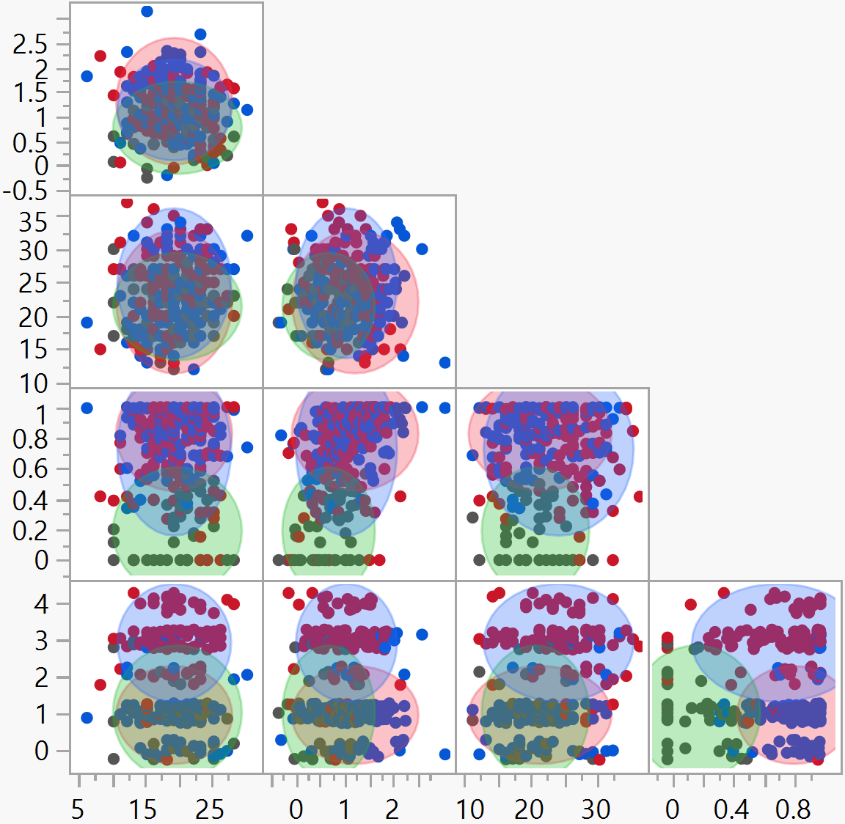

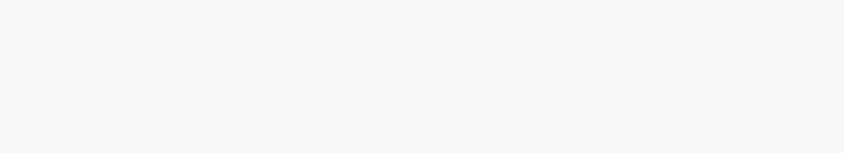

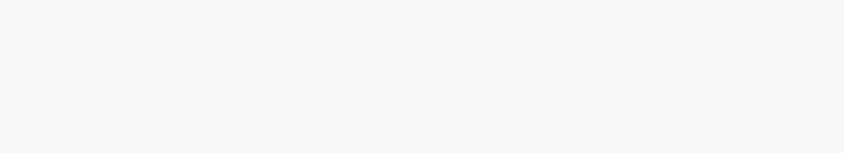

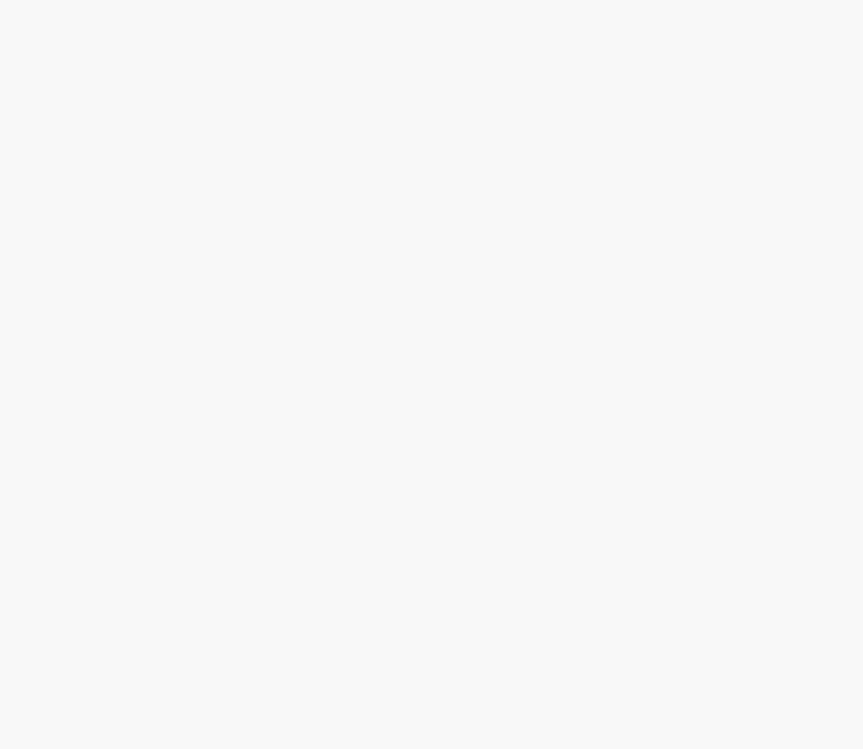


FRS

SHFM

MAGGIC

BCN Bio-HF

CLP

BCN Bio-HF

MAGGIC

SHFM

Caption: Illustration of the relationships between prognostic scores and risk cluster. Each dot represents an observation with its color indicating risk cluster membership. Shaded areas represent the density ellipses. Blue dots indicate cluster 1 (low risk), n=119; Grey dots indicate cluster 2 (moderate risk), n= 44; Red dots indicate cluster 3 (high risk), n= 117. Total subjects, n=280; Each prognostic score was standardized to the same scale (mean=0; SD=1). Ward’s minimum variance method was used for clustering.

SHFM, Seattle Heart Failure Model; FRS, Framingham Risk Score; MAGGIC, Meta-analysis Global Group in Chronic Heart Failure; BCN Bio-HF (Barcelona Bio-Heart Failure Risk Calculator); CLP Risk Score, Cardiac Lipid Panel Risk Score.
