# Supplementary material for: Performance of a cardiac lipid panel compared to four prognostic scores in chronic heart failure
Source: Sci Rep. 2021 Apr 14;11:8164. doi: 10.1038/s41598-021-87776-w (PMC8046832; doi:10.1038/s41598-021-87776-w)
Supplement: Supplementary file 7 — Supplementary Information 7. [file 41598_2021_87776_MOESM7_ESM.docx]

**Supplemental Figure 7: Correlation matrix of baseline clinical characteristics and the CLP biomarkers**


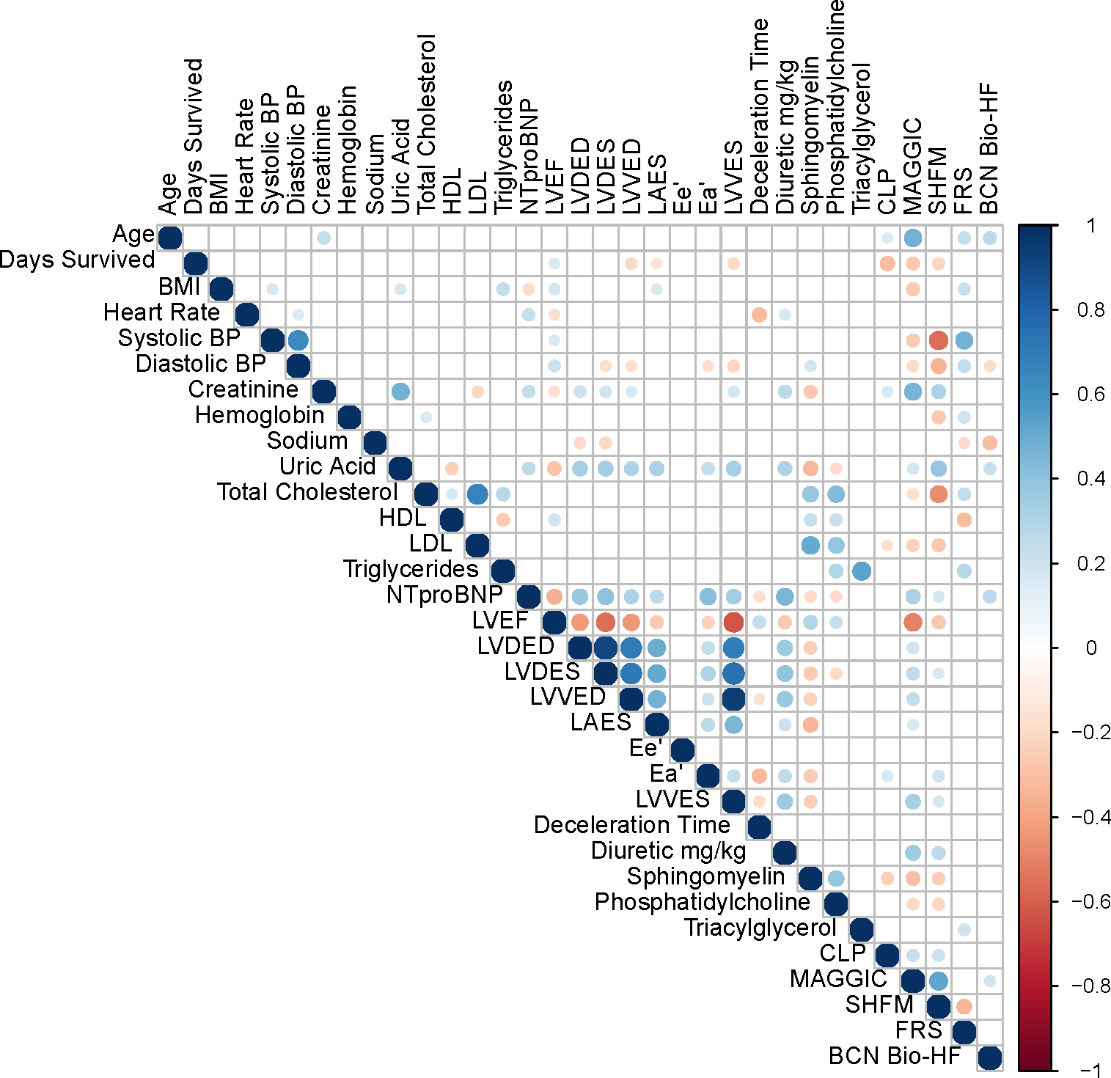


Caption: Colors correspond to the magnitude of Pearson correlations between changes in each pair of variables. Correlation is significant at the 0.01 level (2-tailed). Blank cells are insignificant; n=280. The three CLP biomarkers are: Sphingomyelin (the sum of the 3 isobaric species SM d18:1/23:1, SM d18:2/23:0, and SM d17:1/24:1), Phosphatidylcholine (PC 16:0/18:2), and Triacylglycerol (TAG 18:1/18:0/18:0).

Abbreviations: BCN Bio-HF (Barcelona Bio-Heart Failure Risk Calculator), BMI, body mass index; BP, blood pressure; CLP, cardiac lipid panel; Ee', ratio between early mitral inflow velocity and mitral annular early diastolic velocity; Ea’, ratio of the early (E) to late (A) ventricular filling velocities; FRS, Framingham Risk Score; HDL, high-density lipoprotein; LDL, low-density lipoprotein; LVEF, left ventricular ejection fraction; LVDDED, left ventricular diameter end diastole; LVDDES, left ventricular diameter end systole; LVVED, left ventricular volume end diastole; LVVES, left ventricular volume end systole; LAES, left atrial end systole; MAGGIC, Meta-Analysis Global Group in Chronic Heart Failure; mg/kg, milligrams per kilograms of body weight; NT-proBNP, N-terminal pro–B-type natriuretic peptide; SHFM, Seattle Heart Failure Model
