# Supplementary material for: Performance of a cardiac lipid panel compared to four prognostic scores in chronic heart failure
Source: Sci Rep. 2021 Apr 14;11:8164. doi: 10.1038/s41598-021-87776-w (PMC8046832; doi:10.1038/s41598-021-87776-w)
Supplement: Supplementary file 8 — Supplementary Information 8. [file 41598_2021_87776_MOESM8_ESM.docx]

**Supplemental Table 1: Prognostic Scores and Hazard Ratios for All-Cause Mortality**

| **Score** | **HR (95% CI)** | **p value** |
| --- | --- | --- |
| SHFM | 2.18 (1.18-4.03) | 0.0134 |
| FRS | 0.987 (0.91-1.08) | 0.7669 |
| MAGGIC | 1.2 (1.10-1.28) | <.0001 |
| BCN Bio-HF | 1.1 (0.99-1.23) | 0.0760 |
| CLP | 2.11 (1.53-2.91) | <.0001 |

Caption: Unadjusted Cox proportional hazard models of 3-year all-cause mortality. Total subjects, n=280. Total events, n=30. SHFM (Seattle Heart Failure Model), FRS (Framingham Risk Score), and MAGGIC (Meta-analysis Global Group in Chronic Heart Failure), BCN Bio-HF (Barcelona Bio-Heart Failure Risk Calculator), and Cardiac Lipid Panel Risk Score (CLP)
