# Supplementary material for: Performance of a cardiac lipid panel compared to four prognostic scores in chronic heart failure
Source: Sci Rep. 2021 Apr 14;11:8164. doi: 10.1038/s41598-021-87776-w (PMC8046832; doi:10.1038/s41598-021-87776-w)
Supplement: Supplementary file 9 — Supplementary Information 9. [file 41598_2021_87776_MOESM9_ESM.docx]

**Supplemental Table 2: Harrell's concordance statistics for the discrimination of the five prognostic scores**

| **Model** | **c-statistic (95% CI)** | **Standard Error** | **Concordance** | **Discordance** |  |
| --- | --- | --- | --- | --- | --- |
|  |  |  |  |  |  |
| FRS | 0.52 (0.46-0.59) | 0.0318 | 7266 | 6584 |  |
| SHFM | 0. 60 (0.53-0.67) | 0.0332 | 7487 | 4118 |  |
| MAGGIC | 0.64 (0.58-0.70 | 0.0286 | 10841 | 5938 |  |
| BCN Bio-HF | 0.69 (0.66 -0.72) | 0.0171 | 8435 | 3764 |  |
| CLP | 0.77 (0.74-0.80) | 0.0158 | 11335 | 1847 |  |

Caption: SHFM (Seattle Heart Failure Model), FRS (Framingham Risk Score), and MAGGIC (Meta-analysis Global Group in Chronic Heart Failure), BCN Bio-HF (Barcelona Bio-Heart Failure Risk Calculator), Cardiac Lipid Panel Risk Score (CLP), CI (Confidence Interval). Total subjects, n=280; total events, n=95.
