# Supplementary material for: Performance of a cardiac lipid panel compared to four prognostic scores in chronic heart failure
Source: Sci Rep. 2021 Apr 14;11:8164. doi: 10.1038/s41598-021-87776-w (PMC8046832; doi:10.1038/s41598-021-87776-w)
Supplement: Supplementary file 10 — Supplementary Information 10. [file 41598_2021_87776_MOESM10_ESM.docx]

**Supplemental Table 3: Differences in Uno’s concordance statistic of the five prognostic scores**

| **Model Comparison** | **Estimate** | **Standard Error** | **Chi-Square** | **p-value** |
| --- | --- | --- | --- | --- |
| **FRS vs. MAGGIC** | -0.0629 | 0.0580 | 1.17 | 0.2784 |
| **FRS vs. SHFM** | -0.0200 | 0.0549 | 0.13 | 0.7154 |
| **FRS vs. CLP** | -0.1888 | 0.0493 | 14.69 | <.0001 |
| **MAGGIC vs. SHFM** | 0.0232 | 0.0268 | 0.75 | 0.3861 |
| **MAGGIC vs. CLP** | -0.1259 | 0.0311 | 16.42 | <.0001 |
| **SHFM vs. CLP** | -0.0941 | 0.0277 | 11.53 | 0.0007 |
| **BCN Bio-HF vs. CLP** | -0.0863 | 0.0363 | 5.66 | 0.0173 |
| **MAGGIC vs. BCN Bio-HF** | 0.0232 | 0.0268 | 0.75 | 0.3861 |
| **SHFM vs. BCN Bio-HF** | -0.0740 | 0.0516 | 2.06 | 0.1510 |
| **FRS vs. BCN Bio-HF** | -0.0941 | 0.0277 | 11.53 | 0.0007 |

Caption: SHFM (Seattle Heart Failure Model), FRS (Framingham Risk Score), and MAGGIC (Meta-analysis Global Group in Chronic Heart Failure), BCN Bio-HF (Barcelona Bio-Heart Failure Risk Calculator), and Cardiac Lipid Panel Risk Score (CLP). Total subjects, n=280; total events, n=95.
