# Supplementary material for: Performance of a cardiac lipid panel compared to four prognostic scores in chronic heart failure
Source: Sci Rep. 2021 Apr 14;11:8164. doi: 10.1038/s41598-021-87776-w (PMC8046832; doi:10.1038/s41598-021-87776-w)
Supplement: Supplementary file 11 — Supplementary Information 11. [file 41598_2021_87776_MOESM11_ESM.docx]

|  | **Competing Risk Model** | | | |
| --- | --- | --- | --- | --- |
| **Score** | **CV Mortality** | | **Non-CV Mortality** | |
|  | **HR (95% CI)** | **p value** | **HR (95% CI)** | **p value** |
| SHFM | 1.91 (1.31-2.762) | 0.0007 | 1.01 (0.578-1.752) | 0.9728 |
| FRS | 1.06 (0.961-1.063) | 0.68 | 1.01 (0.95-1.09) | 0.6704 |
| MAGGIC | 1.10 (1.05-1.13) | <.0001 | 1.09 (0.97-1.09) | 0.3924 |
| BCN Bio-HF | 1.04 (0.959-1.109) | 0.3306 | 0.996 (0.887-1.095) | 0.9422 |
| CLP | 2.39 (1.96-3.93) | <.0001 | 1.806 (1.297-2.51) | 0.0004 |

**Supplemental Table 4: Hazard ratios for competing risk models**

Caption: Unadjusted Cox proportional hazard models of 10-year outcome for cardiovascular (CV) mortality with non-CV mortality as a competing event. Total subjects, n=280. Total CV deaths, n=95. Total non-CV death, n=51. SHFM (Seattle Heart Failure Model), FRS (Framingham Risk Score), and MAGGIC (Meta-analysis Global Group in Chronic Heart Failure), BCN Bio-HF (Barcelona Bio-Heart Failure Risk Calculator), and Cardiac Lipid Panel Risk Score (CLP). HR (Hazard ratio), CI (Confidence Interval).
